# Knowledge, attitudes and behaviour of hospital health-care workers regarding influenza A/H1N1: a cross sectional survey

**DOI:** 10.1186/1471-2334-14-208

**Published:** 2014-04-16

**Authors:** Luciana Albano, Anna Matuozzo, Paolo Marinelli, Gabriella Di Giuseppe

**Affiliations:** 1 Department of Experimental Medicine, Second University of Naples, Via Luciano Armanni, 5, 80138 Naples, Italy

**Keywords:** Attitudes, Behaviour, Health-care workers, Influenza A/H1N1, Knowledge

## Abstract

**Background:**

To assess the knowledge, the attitudes, and the behaviour towards influenza A/H1N1 and the vaccination among health-care workers (HCWs).

**Methods:**

A sample of HCWs was selected from a random sample of non-teaching public hospitals, located in the cities of Naples and Avellino (Italy), received a self-administered anonymous questionnaire including questions about socio-demographic characteristics, knowledge on modes of transmission and preventative measures, attitudes and behaviour relating to influenza A/H1N1.

**Results:**

Only 36.1% correctly knew the main modes of transmission, and that HCWs are a risk category and this level of knowledge was significantly higher in HCWs having received information through scientific journals. A higher perceived risk of contracting influenza A/H1N1 has been observed in the HCWs more knowledgeable, in those considering influenza A/H1N1 a serious disease, and in those working in surgical wards. Only 16.7% have received the influenza A/H1N1 vaccination and HCWs with more fear of contracting influenza A/H1N1, those considering vaccine more useful and less dangerous were more likely to receive vaccine.

**Conclusions:**

Education and communication strategies for improving the level of knowledge and for the immunization uptake regarding influenza A/H1N1 HCWs are strongly needed.

## Background

It is well documented that the influenza virus is responsible every year for additional hospitalizations and mortality [1-3] and the recently spread of influenza A/H1N1 has produced preoccupation in several geographic areas. In Italy, as in many other countries, vaccination against the influenza A/H1N1 virus was offered free of charge and health-care workers (HCWs) have been included as one of the first priority groups to receive the vaccination [4]. Indeed, it is well-established that HCWs, because of their close proximity to patients, are recognized to be at risk for both acquiring influenza from patients and transmitting it to patients, and may contribute to hospital influenza outbreaks in health-care facilities [5-7].

HCWs will be expected to play a leadership role in disseminating targeted implementation preventive strategies and education programs aimed at reducing influenza A/H1N1 related morbidity and mortality and in improving the success of promotion campaigns to increase influenza vaccine uptake. Evidence is available that the achievement of these goals varies whether HCWs are knowledgeable, have positive attitudes, and acquire adequate information [8-10]. Although several studies have been conducted worldwide to examine knowledge, attitudes, and behaviour towards influenza A/H1N1 among different categories of HCWs, including general practitioners [11,12], and those working in hospitals such as physicians [13-18], nurses [19,20], and professional support staff [21-24], limited data are available for Italian HCWs [25]. Compared to the study by Bonaccorsi et al., our study evaluated the knowledge about influenza A/H1N1 amongst the HCWs and this findings could have significant implications for information provision and the targeting of future education and communication strategies to this population.

Because a better understanding of the current beliefs of HCWs about the influenza A/H1N1 vaccine may be crucial, the objectives of this cross-sectional survey were to assess the knowledge, the attitudes, and the behaviour towards influenza A/H1N1 and the vaccination among HCWs in several hospitals in a large geographic area of Italy and also the survey presented the opportunity to identify which determinants were associated with knowledge, attitudes, and behaviour.

## Methods

### Enrollment

This cross-sectional study was conducted from November 2009 through January 2010 in the geographic area of Naples and Avellino, Italy in two steps. In the first step, eight public non-teaching hospitals were randomly selected from the list of the 14 hospitals in the area. Next, a simple random sampling was performed in selecting HCWs employed in the hospitals using a random number table, including physicians, nurses, medical technicians and ancillary staff. The research team contacted and presented to the hospital management staff the nature and protocol of the study to obtain permission to carry out the survey in their institution. In the second step, each HCW received a cover letter reporting a detailed study presentation, encouraging responses to the survey in a self-reported form, an informed consent form, a self-administered anonymous questionnaire, and an envelope to return the completed questionnaire. Consent to participate was implied with the completion of the questionnaire. A waiver of signed informed consent was obtained to maintain anonymity.

The sample size was determined before study initiation. The sample size was calculated assuming that 70% of the respondents had an accurate level of knowledge about influenza A/H1N1 in accordance with the literature [16,18], a margin of error of 5%, and a 95% confidence level. Consequently, a sample of 322 HCWs was sought. We decided to be conservative and to increase our sample size to 720 HCWs by anticipating a response rate of 45%.

### Survey instrument

A structured self-administered questionnaire was designed to assess HCWs’ socio-demographic and professional characteristics, knowledge, attitudes, and behaviours about influenza A/H1N1 (see Additional files 1 and 2). Information pertaining socio-demographic and professional characteristics included questions on gender, age, marital status, level of education, professional role, and ward of activity. Knowledge was measured through three questions, one of them using a 3-point Likert-type scale and the response options were “yes”, “do not know”, and “no”. To understand HCWs’ attitudes, five questions were asked, two of them using a 3-point Likert-type scale with options for “agree”, “uncertain”, and “disagree” and 3 items were on a 10-point Likert-type scale, ranging from 1 (unfavorable attitude) to 10 (favorable attitude). The section on behaviours included four questions, 1 of them using a 5-point Likert-type scale ranging from “never” to “always”, and 2 required a yes/no response. In the last section, HCWs were asked about the potential sources of information and whether they felt the need to acquire additional information about influenza A/H1N1.

Questions regarding the definition, modes of transmission, and preventative measures of influenza A/H1N1 were derived from previously published standards [26,27].

### Pilot study

The questionnaire was pre-tested and piloted with a convenience sample of 20 HCWs who were similar in their socio-demographic and professional characteristics to the members of the study population. Based on respondents’ recommendations, some minor rewording and restyling of the questions were incorporated to simplify and improve the final questionnaire.

### Ethics

The Ethical Committee of the Second University of Naples approved the study protocol and the final questionnaire.

### Statistical analysis

Bivariate analyses using *t*-test and chi-square tests have been conducted in order to assess the association between each predictor of interest and the outcomes of interest and those with a *p*-value of 0.25 or better were considered for possible entry in the multivariate linear and logistic regressions equation. Three separate multivariate stepwise logistic and linear regression models were developed for identification of the independent association with the selected predictors and the following outcomes of interest: knowledge of the main modes of transmission and that HCWs are a risk category (no = 0; yes = 1), (Model 1); fear of contracting influenza A/H1N1 (continuous), (Model 2); and having received influenza A/H1N1 vaccine (no = 0; yes = 1), (Model 3). The following independent variables were included in all models: gender, age, marital status, professional role, ward of activity, scientific journals as the source of information, and need of additional information about influenza A/H1N1. The following variables were included: correct knowledge of the main modes of transmission and that HCWs are a risk category, believe that influenza A/H1N1 is a serious disease, and believe that influenza A/H1N1 is a preventable disease in Models 2 and 3. The following variables were included: fear of contracting influenza A/H1N1, believe that influenza A/H1N1 vaccine is useful, and believe that influenza A/H1N1 vaccine is dangerous in Model 3. The stepwise selection procedure with a forward method was used, and a significance level of 0.2 was used as the criterion for variables to enter in the multivariate regression models and 0.4 for variables to remain. Odds ratios (ORs) and their 95% confidence intervals (CIs) were reported as measures of association between predictors and outcomes of interest. All *p*-values of 0.05 or less were considered statistically significant. All analyses were made using the statistical software Stata 10.0 [28].

## Results

Of the 720 HCWs who received the questionnaire, a total of 600 returned the questionnaire yielding a response rate of 83.3%. The socio-demographic characteristics of the respondents are shown in Table 1. Majorities were male, the mean age was 48 years, two-thirds were nurses, and half worked in surgical wards.

**Table 1:** Main socio-demographic characteristics and knowledge about main modes of influenza A/H1N1 transmission of the study population

	***n***	***%***		
***Gender***				
Male	310	52.4		
Female	282	47.6		
***Age group, years***	47.9 ± 8.7 (23–67)*			
≤44	226	39.4		
45-54	194	33.9		
≥55	153	26.7		
***Marital status***				
Married	441	76.3		
Other	137	23.7		
***Professional role***				
Physician	154	27.4		
Nurse	365	65		
Other^a^	43	7.6		
***Ward of activity***				
Medicine	191	33.6		
Surgery	261	45.9		
Other^b^	117	20.5		
***Knowledge***				
	**Yes**		**No**	
	***n***	***%***	***n***	***%***
Droplets after coughing (true)	547	91.2	53	8.8
Droplets after sneezing (true)	530	88.3	70	11.7
Feces and/or urine (false)	77	12.8	523	87.2
Contact with the infected person (true)	423	70.5	177	29.5
Touching mouth after contact with contaminated hands (true)	420	70	180	30
Touching eyes after contact with contaminated hands (true)	346	57.7	254	42.3
Talking (true)	302	50.3	298	49.7
	***n***	***%***		
All correct answers	43	7.2		

*Mean ± Standard deviation (Range).

Numbers for each item may not add up to total number of study population due to missing values.

^**a**^Medical technicians and ancillary staff.

^b^Laboratory, pharmacy, diagnostic imaging.

The survey responses related to knowledge about influenza A/H1N1 of the study participants are reported in Table 1. An high proportion of HCWs correctly knew at least one of the modes of influenza A/H1N1 transmission with frequencies ranging from 91.2% to 50.3%. However, when the knowledge of the main modes of transmission was analyzed combined with the knowledge of the risk categories the percentage was reduced to 36.1. The results of the multivariate logistic regression model showed that this knowledge was significantly higher in HCWs having received information through scientific journals (OR = 1.63; 95% CI = 1.12-2.38) (Model 1 in Table 2).

**Table 2:** Multivariate logistic (1 and 3) and linear (2) regression models results

**Variable**	**OR**	**SE**	**95% CI**	***p *** **value**
**Model 1.** Knowledge of the main modes of transmission about influenza A/H1N1, and that HCWs are a risk category				
Log likelihood = −309.55, *χ*^2^ = 11.34 (3 df), *p* = 0.01				
Having received information through scientific journals	1.63	0.31	1.12-2.38	0.011
Married	1.44	0.34	0.9-2.3	0.13
Need of additional information about influenza A/H1N1	1.35	0.27	0.9-2.01	0.14
**Model 3.** HCWs who had received influenza A/H1N1 vaccine				
Log likelihood = −174.21, *χ*^2^ = 138.87 (5 df), *p* < 0.0001				
Believe that influenza A/H1N1 vaccine is dangerous	0.79	0.05	0.7-0.88	<0.001
Believed that influenza A/H1N1 vaccine is useful	1.51	0.08	1.37-1.69	<0.001
Fear of contracting influenza A/H1N1	1.15	0.07	1.03-1.29	0.013
Wrong knowledge about the main modes of influenza A/H1N1 transmission and that HCWs are a risk category	0.54	0.18	0.29-1.03	0.06
Professional role				
Physician*	1.0	-	-	-
Nurse	0.75	0.22	0.43-1.32	0.32
**Variable**	**Coeff**	**SE**	***t***	***p *** **value**
**Model 2.** Fear of contracting influenza A/H1N1				
F (6,515) = 23.82, *p* < 0.0001, R^2^ = 2.17%, adjusted R^2^ = 2.08%				
Knowledge about the main modes of influenza A/H1N1 transmission and that HCWs are a risk category	0.77	0.2	3.88	<0.001
Believe that influenza A/H1N1 is a serious disease	2.99	0.31	9.64	<0.001
Younger age	−0.03	0.01	−2.7	0.007
Ward activity				
Medicine*	1.0	-	-	-
Surgery	0.4	0.19	2.08	0.04
Believe that influenza A/H1N1 is a preventable disease	−0.33	0.19	−1.75	0.08
Professional role				
Physician*	1.0	-	-	-
Nurse	0.22	0.21	1.07	0.29

*Reference category.

HCWs = Healthcare workers.

Regarding attitudes towards influenza A/H1N1, more than three-quarters of respondents disagreed with the statement that it was a serious illness (88.2%) and more than half of respondents agreed that it was possible to prevent it (53.8%). Attitudes towards risk perception of contracting influenza A/H1N1, measured on a ten-point Likert scale ranging from 1 to 10 with higher scores indicating high fear, showed a mean score for the whole sample of 3.5. The results of the linear regression model indicated that a higher fear of contracting influenza A/H1N1 was observed in HCWs, more knowledgeable, in those considering influenza A/H1N1 a serious disease, and in those working in surgical wards when medical wards were chosen as the reference category (Model 2 in Table 2). The mean value of respondents’ attitude regarding the utility of the vaccination in order to prevent the influenza A/H1N1 and the dangerousness of the vaccine, measured on a ten-point Likert scale ranging from 1 to 10 with higher scores indicating more positive attitudes, was respectively 4.5 and 5.7.

The vast majority of the HCWs self-reported that they perform the disinfection in their working activity. However, among these HCWs, appropriate procedures were reported with different frequencies ranging from 31% for using mask for visiting patients to 86.1% for using new gloves after each task; particularly, 78.2% washed their hands between a patient and the other.

The responses related to the vaccine acceptance indicated that only 16.7% of the sample self-reported that they have received it. However, among those HCWs who had been not vaccinated, 43.6% and 1.4% would still not consider the vaccination in the future and intended to have the vaccine in the future, respectively. Barriers for not having received the vaccine include fear of adverse effects (31.1%), low awareness of the severity of influenza (20.5%), and beliefs that the HCW was not at risk for influenza (8.3%) and that the vaccine had a low efficacy (6.4%). The results of the multivariate logistic regression model along with the ORs and 95% CIs indicated that HCWs with more fear of contracting influenza A/H1N1 (OR = 1.15; 95% CI = 1.03-1.29), those considering vaccine more useful (OR = 1.51; 95% CI = 1.37-1.69) and less dangerous (OR = 0.79; 95% CI = 0.7-0.88) were more likely to receive the vaccine (Model 3 in Table 2).

Almost all respondents stated that they were exposed to information about influenza A/H1N1 and the majority acquired knowledge from public-media (63.5%), followed by health-care professionals (47.1%), and the Internet (45%). When asked whether they would like to learn more about influenza A/H1N1, only one-third (32.8%) answered affirmatively.

## Discussion and conclusions

The results reported in this survey offer a first insight regarding HCWs’ knowledge, attitude, and practice toward influenza A/H1N1 infection in Italy.

The research findings revealed a general widespread lack of knowledge regarding at least one of the modes of influenza A/H1N1 transmission, with frequencies ranging from 91.2% to 50.3%. This finding was remarkable lower than one observed in a survey in a multispecialty teaching hospital in India, because all resident doctors and more than 90% of nurses knew that influenza was caused by a virus that was transmitted by the droplet method [18]. It is concerning this low level of knowledge and it was surprising especially given that HCWs need to be aware of how they may transmit and acquire influenza during their working activity. Thus, the findings suggest the urgent need for educational programs that explicitly must increase the level of knowledge among this group. In the final multivariable model, the results lead to the conclusion that scientific journals as the source of information play a significant role in HCWs gaining knowledge about influenza A/H1N1. Those HCWs who had received information through scientific journals were much more likely to answer the knowledge question correctly. This finding parallels other previously conducted research that observed how this way of communicating information has an important impact on HCWs’ knowledge [14,29]. The survey instrument contained attitude statements related to perceptions of the severity of influenza, susceptibility to it, and the benefits incurred in undertaking the vaccine. The perceived risk of contracting influenza A/H1N1 was low with a mean score, on a scale from 1 to 10, of 3.5. In a study conducted in a tertiary teaching hospital in Greece, more than half of HCWs experienced moderately high levels of worry about the pandemic [21]. It is interesting to note that the current study’s data demonstrate that the specialty was associated with HCWs’ beliefs related to influenza A/H1N1, notably that HCWs working in surgical wards were more likely than those working in medical wards to have a higher fear of contracting influenza A/H1N1.

The current study also sheds light on HCWs’ influenza A/H1N1 vaccine coverage and interest in receiving the vaccine. A small proportion of this population was vaccinated (16.7%) and intent to receive the vaccine (1.4%) which is consistent with a previous survey finding conducted in Spain with a vaccination coverage of 16.5% [15] and with data observed in studies conducted in Italy, 15% [25]. By contrast, these frequencies were remarkably low compared to other similar studies. Indeed, in a sample of HCWs in a governmental hospital located in Turkey a total of 23.1% received the pandemic influenza A/H1N1 vaccine [22]. In a pediatric oncology referral center in the United States 75.2% of HCWs reported receiving 2009 H1N1 influenza vaccine [30]. The uptake rates for monovalent 2009 pandemic H1N1 vaccine among hospital-based doctors, nurses, and allied HCWs in Singapore and United Kingdom were 36.2% and 41.3%, respectively [29]. The vaccination rate against pandemic influenza among Dutch general practitioners was 85% [11]. The coverage rate in a French Teaching Hospital was 36.5% [17]. Lower frequencies of HCWs receiving the vaccine have been observed in Spain with 14.8% [23]. Information needs to be communicated properly to prevent the spread of inaccurate or incomplete information about the safety and efficacy of the influenza A/H1N1 vaccine. Unaddressed concerns may have an impact, particularly given the fact that HCWs are one of the primary target groups for the influenza A/H1N1 vaccine as they are exposed to the virus. Therefore, it is important that messages and promotional campaigns are provided to this target population to emphasize directly the role of the vaccination in terms of personal and patient protection and to eliminate misconceptions about the vaccine in order for HCWs to make appropriate choices of influenza A/H1N1 prevention strategies. The results of the multivariate analysis showed a significant association between having received the vaccine with a high perceive risk of contracting influenza A/H1N1, considering vaccine more useful and less dangerous, and thus the prime focus should be to enhance this knowledge. Furthermore, information-seeking behaviour was rather high, with almost all actively sought information. It is crucial to have access to reliable information sources, and it should be noted that the non-scientific, such as the media, was the main source. This is in accordance with previously conducted studies [22,24].

Although the findings of this study help illuminate the knowledge, attitudes, and practices towards influenza A/H1N1 infection in Italy, it is important to underline that the interpretation of the results should be assessed in the context of potential identified limitations in the study design and execution. First of all, it should be noted that, because this study has a cross-sectional design, the relationship between the predictor variables and the dependent variables should not be taken as cause-and-effect relationship but the study is able only to describe general associations. Second, as with any survey based on self-administered questionnaire, self-reported information from which the analysis and interpretations are based may not be entirely accurate, mainly because we have encountered under-reporting of risky behaviours, and, therefore, should be viewed with caution. This may limit the reliability of the findings because of the possibility that HCWs could give a more positive picture than would be revealed by other data collection methods. Third, HCWs who had been already vaccinated may be more likely to participate. However, coverage with the H1N1 vaccine in this sample of HCWs was similar to levels documented for all HCWs in Italy [31]. Despite the limitations identified, we believe that the findings have important implications for influenza A/H1N1 prevention and future research.

The findings from the survey have implications for the development of influenza A/H1N1 education and communication strategies for HCWs that provide specific and detailed information suitable to the requirements of improving the level of knowledge about this issue and of optimizing immunization uptake.

## Competing interests

The authors declare that they have no competing interests.

## Authors’ contributions

LA participated in the design of the study, contributed to the data analysis and interpretation; AM participated in the design of the study, collected the data, and contributed to the data analysis; PM contributed to the data analysis and interpretation; GDG participated in the design of the study, was responsible for the data statistical analysis and interpretation, and wrote the article. All authors have read and approved the final version of the manuscript.

## Pre-publication history

The pre-publication history for this paper can be accessed here:

http://www.biomedcentral.com/1471-2334/14/208/prepub

## Supplementary Material

Additional file 1 Questionnaire - Original version. Click here for file

Additional file 2 Questionario - Versione originale. Click here for file
