# Supplementary material for: Knowledge, attitudes and behaviour of hospital health-care workers regarding influenza A/H1N1: a cross sectional survey
Source: BMC Infect Dis. 2014 Apr 16;14:208. doi: 10.1186/1471-2334-14-208 (PMC4021506; doi:10.1186/1471-2334-14-208)
Supplement: Additional file 1 — Questionnaire - Original version. [file 1471-2334-14-208-S1.doc]

## DEMOGRAPHIC AND PRACTICE CHARACTERISTICS

**A1.** How old were you on your last birthday? _________

**A2.** Gender (don’t ask) ٱ Male ٱ Female

**A3.** What is your marital status? ٱ Married ٱ Single (never married) ٱ Other

**A4.** How many children do you have? ___

**A5.** What is your education level? ______________________

**A6.** How many years have you been working in a poultry industry/farm?___________

**A7.** How many hours per day do you work in the poultry farm?___________

**A8.** What is your type of work in the poultry farm? _____________________________________________

### KNOWLEDGE

This section of the interview is designed to explore your knowledge related to Avian Influenza (AI).

**B1.** How do you define AI?_______________________________________________________________

I will read to you some statements regarding AI. For each statement, please give me a yes or no answer.

**B2.** AI is transmitted by Animal-to-human ٱ Yes ٱ No

Animal-to-animal ٱ Yes ٱ No

Human-to-human ٱ Yes ٱ No

Environment-to-human ٱ Yes ٱ No

Eating uncooked poultry foods ٱ Yes ٱ No

Eating cooked poultry foods ٱ Yes ٱ No

Eating uncooked eggs ٱ Yes ٱ No

Eating cooked eggs ٱ Yes ٱ No

Touching uncooked poultry foods ٱ Yes ٱ No

Touching cooked poultry foods ٱ Yes ٱ No

Touching uncooked eggs ٱ Yes ٱ No

Touching cooked eggs ٱ Yes ٱ No

Saliva, nasal secretions, feces, and fomite of infected birdsٱ Yes ٱ No

Poultry ٱ Yes ٱ No

Wild birds ٱ Yes ٱ No

Rabbits ٱ Yes ٱ No

Cows ٱ Yes ٱ No

**B3.** Individuals at risk for contracting AI are

Poultry workers ٱ Yes ٱ No

Butchers ٱ Yes ٱ No

Veterinarians ٱ Yes ٱ No

Hunters ٱ Yes ٱ No

I am going to read a list of measures concerning prevention of AI and for each of them I would like you to tell me whether you agree, are uncertain or disagree.

**B4.** Wash hands with soap and water ٱAgree ٱUncertain ٱDisagree

**B5.** Facemask ٱAgree ٱUncertain ٱDisagree

**B6.** Gloves ٱAgree ٱUncertain ٱDisagree

**B7.** Outer garments ٱAgree ٱUncertain ٱDisagree

**B8.** Boots or boot covers ٱAgree ٱUncertain ٱDisagree

**B9.** Eye-protection ٱAgree ٱUncertain ٱDisagree

**B10.**Wash and disinfect utensils ٱAgree ٱUncertain ٱDisagree

**B11.**Wash and disinfect surfaces ٱAgree ٱUncertain ٱDisagree

###### ATTITUDES

I would like to know your attitudes towards AI. Try to answer the following questions as truthfully as possible.

**C1.** AI may be prevented ٱ Agree ٱ Uncertain ٱ Disagree

**C2.** AI is a serious disease ٱ Agree ٱ Uncertain ٱ Disagree

**C3.** How would you rate your fear of getting AI on a 1 to 10 scale with 1 meaning no fear at all and 10 very much fear?

1 2 3 4 5 6 7 8 9 10

No fear at all Very much fear

**C4.** How would your colleagues/familiars rate their fear of getting AI on a 1 to 10 scale with 1 meaning no fear at all and 10 meaning very much fear?

1 2 3 4 5 6 7 8 9 10

No fear at all Very much fear

###### BEHAVIORS

I am going to ask you some questions which are designed to gather information about your behavior.

**D1.** In the past three months have you modified your working habits for fear of getting AI?

ٱ No ٱ Yes (Please specify__________________________________)

**D2.** In your activity how often do you use each of the following preventive measures to avoid spreading of the AI virus through food (after reading each item ask “Would you say never, rarely, sometimes, often, always?”)?

**D2-1.** Outer garments ٱ Always ٱ Often ٱ Sometimes ٱ Rarely ٱ Never

**D2-2.** Gloves ٱ Always ٱ Often ٱ Sometimes ٱ Rarely ٱ Never

**D2-3.** Facemask ٱ Always ٱ Often ٱ Sometimes ٱ Rarely ٱ Never

**D2-4.** Eye protection ٱ Always ٱ Often ٱ Sometimes ٱ Rarely ٱ Never

**D2-5.** Boots or boot covers ٱ Always ٱ Often ٱ Sometimes ٱ Rarely ٱ Never

**D3.** How often do you wash your hands?

ٱ Always ٱ Often ٱ Sometimes ٱ Rarely ٱ Never (go to D5)

**D4.** How do you wash your hands? ٱ With water ٱ With water and soap ٱ With disinfectant

**D5.** How often do you wash and disinfect surfaces and utensils?

ٱ Always ٱ Often ٱ Sometimes ٱ Rarely ٱ Never

E. INFORMATION

**E1.** From which of the following sources of information do you receive AI education? (more than one answer if possible)

ٱ None ٱ Mass-media ٱ Friend/Familiar ٱ Employer ٱ Health professional ٱ Other (specify _________)

E2. Do you feel you need more information about AI? ٱ Yes ٱ No
