# Supplementary material for: Knowledge, attitudes and behaviour of hospital health-care workers regarding influenza A/H1N1: a cross sectional survey
Source: BMC Infect Dis. 2014 Apr 16;14:208. doi: 10.1186/1471-2334-14-208 (PMC4021506; doi:10.1186/1471-2334-14-208)
Supplement: Additional file 2 — Questionario - Versione originale. [file 1471-2334-14-208-S2.doc]

**A. INFORMAZIONI ANAGRAFICHE E PROFESSIONALI**

**A1.** Sesso: **□** M **□** F **A2.** Anno di nascita: _______ **A3.** Stato civile: **□** celibe/nubile **□** sposato/a **□** altro ___

**A4.** Qual è il più avanzato titolo di studio che ha conseguito?

**□** nessuno **□** elementare **□** media-inferiore **□** media-superiore **□** laurea

**A5.** Qual è il Suo ruolo professionale? □ infermiere □ medico **□**altro ______________

**A6.** In quale reparto lavora?: ______________________ **A7.** Da quanti anni lavora in questo reparto? ______

**B. CONOSCENZE**

**B1.** Che cos’è l’influenza A/H1N1?

□ malattia dei maiali □ malattia trasmessa dai maiali □ malattia di origine animale trasmessa tra uomo ed uomo □ non so

**B2.** Come si può trasmettere l’influenza A/H1N1?

Sì No Non so

- contatti con feci e/o urine contaminate **□** **□** **□**

- contatto con persone infette **□** **□** **□**

- goccioline emesse con la tosse **□** **□** **□**

- goccioline emesse con lo starnuto **□** **□** **□**

- parlando **□** **□** **□**

- toccando gli occhi con mani non lavate **□** **□** **□**

- toccando la bocca con mani non lavate **□** **□** **□**

- altro________________________________________________________________________________

**B3.** Quali sono le categorie di persone che sono più a rischio di contrarre l’influenza A/H1N1? (**anche più di una risposta**)

□ bambini □ giovani sani tra 18 e 27 anni □ persone a rischio, di età compresa tra 6 mesi e 65 anni

□ anziani □ donne in gravidanza al II/III trimestre □ soggetti con patologie croniche

(es. broncopatia cronica, diabete)

□ militari □ personale della protezione civile □ operatori sanitari (es.medici, infermieri, etc.)

□ donatori di sangue □ personale delle forze di pubblica sicurezza (es. polizia, etc.)

□ altro________________________________________________________________________________

**C. ATTITUDINI**

**D’accordo Incerto In disaccordo**

**C1.** Ritiene l’influenza A/H1N1 una malattia grave? **□** **□** **□**

**C2.** Ritiene l’influenza A/H1N1 una malattia prevenibile? **□** **□** **□**

**C3.** In una scala da 1 a 10, indicare con un numero, quanta paura ha di contrarre l’influenza A/H1N1? (**1 indica nessuna paura e 10 molta paura**)

1 2 3 4 5 6 7 8 9 10

**nessuna paura molta paura**

**C4.** In una scala da 1 a 10, indicare con un numero, quanto ritiene utile il vaccino per prevenire l’influenza A/H1N1? (**1 indica nessuna utilità e 10 molta utilità**)

1 2 3 4 5 6 7 8 9 10

**nessuna utilità molta utilità**

**C5.** In una scala da 1 a 10, indicare con un numero, quanto ritiene pericoloso il vaccino per prevenire l’influenza A/H1N1? (**1 indica nessuna preoccupazione e 10 molta preoccupazione**)

1 2 3 4 5 6 7 8 9 10

**nessuna preoccupazione molta preoccupazione**

**D. COMPORTAMENTI**

**D1.** Indicare con quale frequenza:

- usa la mascherina nel visitare i pazienti □ sempre □ spesso □ qualche volta □ raramente □ mai

- lava le mani tra paziente e paziente □ sempre □ spesso □ qualche volta □ raramente □ mai

- cambia i guanti dopo ogni attività □ sempre □ spesso □ qualche volta □ raramente □ mai

- utilizza disinfettanti specifici □ sempre □ spesso □ qualche volta □ raramente □ mai

per il lavaggio delle mani

**D2.** Ha modificato, negli ultimi mesi, i suoi comportamenti in merito all’utilizzo di mascherine, guanti e disinfettanti?

□ no □ sì che cosa ha modificato?____________________________________________________

**D3.** Si è vaccinato o si vaccinerebbe contro l’influenza A/H1N1?

□ Sì, mi sono vaccinato □ Sì, mi vaccinerei □ No, non mi sono vaccinato □ No, non mi vaccinerei

**( indicare uno o più motivi )**  **( indicare uno o più motivi )**

| □ per la non gravità della malattia  □ ho già contratto l’influenza A/H1N1 □ per la mancata disponibilità del vaccino □ per la scarsa efficacia del vaccino  □ per timore degli effetti collaterali del vaccino  □ per miei precedenti reazioni avverse ai vaccini □ per l’esistenza di trattamenti alternativi (antivirali) □ perché non sono un soggetto a rischio  □ altro (specificare______________________) |
| --- |

| □ per la gravità della malattia □ per l’efficacia del vaccino **□** per la sicurezza del vaccino □ per proteggere la mia famiglia □ per proteggere i miei pazienti □ per proteggere me stesso  **□** per la gravità delle complicanze □ perché raccomandato dal Ministero della Salute  □ altro (specificare__________________) |
| --- |

**D4.** A chi non ha consigliato/non raccomandato e a chi non consiglierebbe/non raccomanderebbe il vaccino contro l’influenza A/H1N1?

| □**non l’ho raccomandato a: (anche più di una risposta)** | □ **non l’ho consigliato a:**  **(anche più di una risposta)** | □ **non lo raccomanderei a**  **(anche più di una risposta)** | □ **non lo consiglierei a:**  **(anche più di una risposta)** |
| --- | --- | --- | --- |
| □ nessuno  **(indicare il/i motivo/i)**  **_________________________**  _____________________ | □ nessuno  **(indicare il/i motivo/i)**  **_________________________**  ______________________ | □ nessuno  **(indicare il/i motivo/i)**  **_________________________**  ______________________ | □ nessuno  **(indicare il/i motivo/i)**  **_________________________**  _____________________ |
| □ famiglia  **(indicare il/i motivo/i)**  **_________________________**  _____________________ | □ famiglia  **(indicare il/i motivo/i)**  **_________________________**  ______________________ | □ famiglia  **(indicare il/i motivo/i)**  **_________________________**  ______________________ | □ famiglia  **(indicare il/i motivo/i)**  **_________________________**  _____________________ |
| □ conoscenti  **(indicare il/i motivo/i)**  **_______________________**  **_____________________________** | □ conoscenti  **(indicare il/i motivo/i)**  **_____________________________**  **______________________________** | □ conoscenti  **(indicare il/i motivo/i)**  **_____________________________**  **______________________________** | □ conoscenti  **(indicare il/i motivo/i)**  **____________________________**  **_____________________________** |
| □ pazienti  **(indicare il/i motivo/i)**  ____________________  _____________________ | □ pazienti  **(indicare il/i motivo/i)**  ______________________  ______________________ | □ pazienti  **(indicare il/i motivo/i)**  ______________________  ______________________ | □ pazienti  **(indicare il/i motivo/i)**  _____________________  _____________________ |
| □ altro  **(indicare il/i motivo/i)**  _______________________  _______________________ | □ altro  **(indicare il/i motivo/i)**  ________________________  ________________________ | □ altro  **(indicare il/i motivo/i)**  ________________________  ________________________ | □ altro  **(indicare il/i motivo/i)**  _______________________  _______________________ |

**E. INFORMAZIONI**

**E1.** Da quali fonti ha acquisito informazioni sulla influenza A/H1N1? **(indicare anche più di una risposta)**

□ nessuna □ medici □ TV/giornali □ riviste scientifiche □ amici/parenti □ internet

□ altro (**specificare**__________________________________________________________)

**E2.** Ritiene di aver bisogno di ulteriori informazioni sull’influenza A/H1N1? □ no □ sì
